# Bewältigung infektiologischer Gefahrenlagen in deutschen Häfen: Entwicklung eines standardisierten Konzepts im Verbundforschungsprojekt „Gesunde Häfen, gemeinsam stark (GESA)“

**DOI:** 10.1007/s00103-025-04143-0

**Published:** 2025-10-23

**Authors:** Marie Frese, Matthias Boldt, Lena Ehlers, Maria an der Heiden, Scarlett Kleine-Kampmann, Marcus Oldenburg, Juliane Seidel, Jette Zimmermann, Jan Heidrich, Volker Harth

**Affiliations:** 1 Zentralinstitut für Arbeitsmedizin und Maritime Medizin (ZfAM), Universitätsklinikum Hamburg-Eppendorf (UKE), Seewartenstraße 10, 20359 Hamburg, Deutschland; 2 Hamburg Port Health Center (HPHC), Institut für Hygiene und Umwelt der Freien und Hansestadt Hamburg (HU), Hamburg, Deutschland; 3 Fachgebiet 31: ÖGD-Kontaktstelle Krisenmanagement, Ausbruchsuntersuchungen und Trainingsprogramme, Abteilung für Infektionsepidemiologie, Robert Koch-Institut, Berlin, Deutschland

## Einleitung

Häfen stellen als Dreh- und Angelpunkte des internationalen Handels- und Reiseverkehrs potenzielle Eintrittspforten für infektiologische Gefahren dar. Gleichzeitig erhöhen die Globalisierung und der stetig wachsende Schiffsverkehr das Risiko von Krankheitsausbrüchen, die sich schnell über Kontinente hinweg ausbreiten können. Spezifische räumliche und hygienische Bedingungen an Bord wie die Kombination aus einem beengten Zusammenleben und regelmäßigem Wechsel von Besatzungen und Passagieren können dazu beitragen, dass Infektionen sich verbreiten und schwer kontrollierbar werden. Insbesondere auf Kreuzfahrtschiffen kommt es immer wieder zu Infektionsausbrüchen [1, 2]. Darüber hinaus birgt diese Dynamik ein Risiko für die öffentliche Gesundheit, da Infektionserkrankungen am Hafen auf die lokale Bevölkerung und durch Reiserückkehrende bundesweit übertragen werden können. Hieraus ergeben sich vielschichtige Herausforderungen für den Öffentlichen Gesundheitsdienst (ÖGD). Diese reichen von Herausforderungen beim konkreten Ausbruchsmanagement bis hin zu politischen Spannungsfeldern, beispielsweise bei Einreise- und Quarantänebestimmungen [3].

Grenzübergangsstellen, sogenannte Points of Entry (PoEs), können eine zentrale Rolle bei der Prävention und Eindämmung von Krankheitsausbrüchen einnehmen. Die Internationalen Gesundheitsvorschriften (IGV) der Weltgesundheitsorganisation (WHO) definieren in Anlage 1 Teil B hierfür verbindliche Kernkapazitäten, welche die IGV-designierten Häfen vorhalten müssen, um adäquat auf gesundheitliche Bedrohungslagen reagieren und eine Ausbreitung von Infektionskrankheiten zu verhindern [4]. Diese Kernkapazitäten umfassen jederzeit verfügbare Strukturen und Ressourcen, wie eine funktionierende Kommunikation, ausreichend qualifiziertes Personal, sowie den Zugang zu medizinischen Diensten, einschließlich Diagnoseeinrichtungen. Außerdem betreffen sie spezifische Anforderungen für den Ereignisfall, insbesondere bei gesundheitlichen Notlagen Internationaler Tragweite (GNIT). Hierzu zählen die Verfügbarkeit von Notfallplänen, Kapazitäten zur Untersuchung und Versorgung betroffener Reisender, geeignete Räumlichkeiten zur Befragung krankheitsverdächtiger Personen sowie die Durchführung medizinischer Ein- und Ausreisekontrollen [5].

In Deutschland sind gemäß dem IGV-Durchführungsgesetz von 2013 5 Häfen (Bremen/Bremerhaven, Hamburg, Kiel, Rostock und Wilhelmshaven) benannt, definierte Kapazitäten nach Anlage 1B der IGV der WHO umzusetzen [6]. Der Stand der Implementierung der IGV wird im Rahmen des IGV-Monitoring and Evaluation Framework der WHO evaluiert, unter anderen durch eine verpflichtende jährliche Selbsteinschätzung der Mitgliedsstaaten und durch freiwillige Joint External Evaluations (JEE) unter Leitung der WHO zur Identifikation von Stärken und Entwicklungspotenzialen [7]. Eine im Jahr 2019 in Deutschland erstmalig durchgeführte JEE, die jedoch pandemiebedingt nicht mit einer Berichtsveröffentlichung abgeschlossen werden konnte, verdeutlichte hierbei den Handlungsbedarf, den Allgefahrenansatz stärker zu berücksichtigen und über die IGV-benannten Häfen hinauszudenken. Konkret sollten die Aus- und Fortbildung gestärkt, die Notfallplanung optimiert und einheitliche Abläufe sowie ein überregionaler Austausch etabliert werden.

### Das Projekt GESA

Um die Kompetenzen der deutschen IGV-Häfen im Hinblick auf den Umgang mit Gesundheitsgefahren im und durch den maritimen Transportsektor zu stärken, wurde vom Zentralinstitut für Arbeitsmedizin und Maritime Medizin (ZfAM) am Universitätsklinikum Hamburg-Eppendorf (UKE) und dem Hamburg Port Health Center (HPHC) des Instituts für Hygiene und Umwelt (HU) der Freien und Hansestadt Hamburg das Verbundforschungsprojekt „Gesunde Häfen, gemeinsam stark (GESA)“ initiiert. Die Zusammenarbeit aus Wissenschaft und Öffentlichem Gesundheitsdienst schafft Synergien und hat sich bereits im Rahmen des Projektes „Adaptives Resilienz Management im Hafen (ARMIHN)“ bewährt [8]. Der Verbund kooperiert dabei mit den Hafenärztlichen Diensten (HÄD) der 5 IGV-designierten Häfen. Darüber hinaus wurde die Abteilung für Infektionsepidemiologie des Robert Koch-Instituts (RKI) miteinbezogen.

Die Projektlaufzeit erstreckte sich von Mai 2023 bis Juli 2025. Gefördert wurde das Projekt durch das Bundesministerium für Gesundheit (BMG) im Rahmen der Förderbekanntmachung „Strukturelle Stärkung und Weiterentwicklung des Öffentlichen Gesundheitsdienstes (ÖGD)“ (Förderkennzeichen ZMI5-2523SGW00A/B).

Ziel des Projektes ist die Entwicklung eines Konzeptes für übergreifende Strukturen zum Umgang mit infektiologischen Gefahrenlagen im maritimen Sektor. Dazu wurden unter anderem folgende Fragestellungen entwickelt:

- Welche strukturellen Bedarfe bestehen in den 5 Hafenärztlichen Diensten der IGV-designierten Häfen in Deutschland? Wo gibt es Überschneidungen?
- Welche Tätigkeiten der Hafenärztlichen Dienste können harmonisiert werden? Wie kann eine solche Harmonisierung ausgestaltet werden?
- Welche Aus- und Fortbildungsbedarfe sind für Akteure des Öffentlichen Gesundheitsdienstes mit Bezug zu IGV-Häfen vorhanden? Wie können diese gestaltet und etabliert werden?

**Abb. 1:**
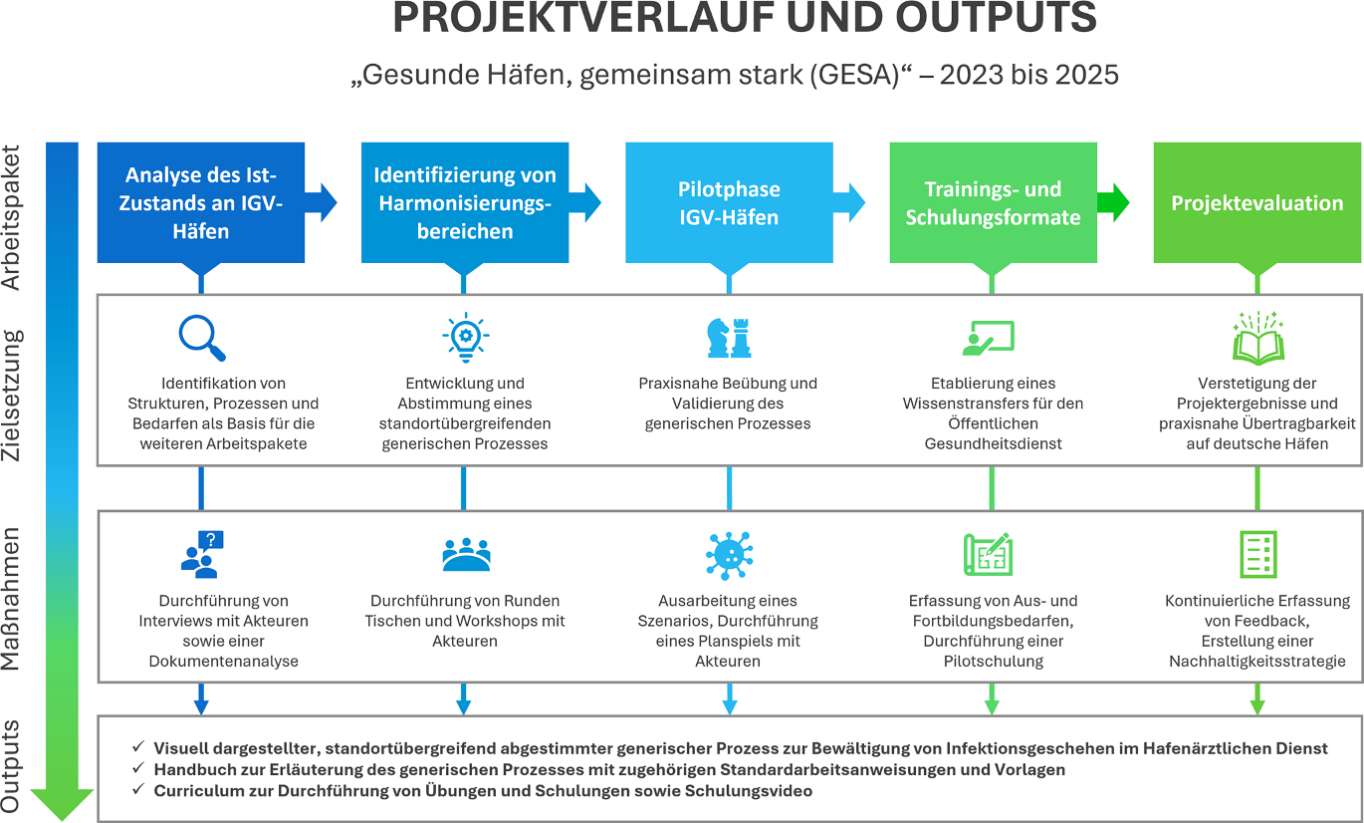
Überblick über den Verlauf des GESA-Projektes (Arbeitspakete, Zielsetzung, durchgeführte Maßnahmen) und die Ergebnisse (Outputs) des Forschungsvorhabens. (*IGV* Internationale Gesundheitsvorschriften)

Zur Bearbeitung dieser Fragestellungen wurde ein Studiendesign mit folgenden Arbeitspaketen entwickelt: Analyse des Ist-Zustands an IGV-Häfen, Identifizierung von Harmonisierungsbereichen, Pilotphase IGV-Häfen, Trainings- und Schulungsformate sowie Projektevaluation. Das Studiendesign mitsamt seinen Zielsetzungen, durchgeführten Maßnahmen und Ergebnissen wird in Abb. 1 zusammengefasst dargestellt. Der vorliegende Projektbericht gibt einen Überblick über die durchgeführten Maßnahmen sowie die Ergebnisse, aufgeteilt in die Beschreibung der Erhebung des Ist-Zustands an IGV-Häfen, die Schritte zur Entwicklung eines generischen Prozesses zur Bewältigung von infektiologischen Ereignissen sowie die Darstellung des Wissenstransfers für Mitarbeitende des Öffentlichen Gesundheitsdienstes auf Grundlage der Ergebnisse.

## Erhebung des Ist-Zustands an deutschen IGV-Häfen

### Durchführung von Interviews

Zu Beginn des Projektes stand eine Ist-Analyse hinsichtlich der Vorbereitungen auf infektiologische Gefahrenlagen in den 5 deutschen IGV-Häfen. Hierbei wurden 35 Interviews mit Mitarbeitenden folgender Organisationen durchgeführt: Hafenärztliche Dienste, Hafenbehörden, Terminalbetreiber, Seemannsmissionen, Feuerwehren, (Elb‑)Lotsen, Wasserschutz- und Bundespolizei, Zollämter und das Havariekommando. Inhalte der Interviews waren Tätigkeiten und Zuständigkeiten bei Infektionsgeschehen an Bord sowie bisherige Erfahrungen und Bedarfe. Eine detaillierte Beschreibung der Durchführung und der Ergebnisse findet sich an anderer Stelle [9]. Zusammengefasst zeigen die Ergebnisse, dass sich die Abläufe und die Zusammenarbeit zwischen den verschiedenen institutionellen Akteuren insbesondere durch die COVID-19-Pandemie weitestgehend eingespielt haben:

„Also da muss man sagen, das ist so einer der wenigen Vorteile von Corona, dass die Abläufe bei infektiösen Notfällen sich extrem verbessert haben“ (Hafenärztlicher Dienst).

Der Bedarf an persönlichem und stetigem Austausch zwischen den unterschiedlichen Akteuren auch nach der Pandemie wurde von vielen Befragten hervorgehoben. Dies sollte auch in gemeinsamen Übungen erfolgen, um die Abläufe insbesondere an Schnittstellen zu trainieren und somit in Einsatzlagen effektiv zusammenarbeiten zu können.

„Man kann das ja alles auf dem Plan aufmalen. Wir können sagen, da ist der Liegeplatz und hier könnt ihr euer Zelt aufbauen. Aber das vielleicht ein bisschen genauer durchzuspielen, wäre sicherlich hilfreich, und das wäre dann … mit den Akteuren hier vor Ort“ (Hafenbetreiber).

In den Interviews wurden zudem relevante Themenfelder für Trainings und Schulungen identifiziert. Diese ließen sich den Oberkategorien „Maßnahmen und Abläufe“ sowie „Gefährdungsbeurteilung und Prävention“ zuordnen. Eine akteursübergreifende Übersicht dieser Themen ist in Tab. 1 dargestellt. Mehrfach wurde auf die Relevanz der Vertrautheit mit spezifischen Prozessen innerhalb der eigenen Organisation sowie in der Zusammenarbeit mit externen Akteuren eingegangen. Auch Schulungsbedarfe hinsichtlich der rechtlichen Rahmenbedingungen wurden geäußert, insbesondere im Hinblick auf die internationalen Gesundheitsvorschriften und Kernkapazitäten. Akteure ohne medizinische Expertise äußerten vermehrt den Bedarf an Hintergrundinformationen über Infektionskrankheiten, wie Übertragungswege und Hygieneverhalten.

**Tab. 1:** Themenfelder für Aus- und Fortbildung im Infektionsschutz nach Einschätzung des Hafenärztlichen Dienstes und weiterer Akteure

Kategorie	Themen für Aus- und Fortbildung
Maßnahmen und Abläufe	Beschreibung von Strategien für resiliente Häfen während Pandemien
	Beschreibung spezifischer Aufgaben und Zuständigkeiten
	Maßnahmen und Abläufe an Bord, je nach Infektion
	Logistik bei Evakuierung/Absonderung von Passagieren/Crew
	Unterschiede und Einsatztaktiken nach Schiffstypen (beispielsweise Kauffahrtei- und Passagierschiffe)
	Verständnis von Gesundheitsmaßnahmen
	Sensibilisierung für die Aufgaben anderer Akteure
	Interdisziplinäre Zusammenarbeit und Schnittstellen
	Kommunikationswege und Meldeketten
	Definition klarer Kriterien und Parameter für Gesundheitslagen/Massenanfall an Erkrankten
	Modellierung und Simulation von Szenarien (beispielsweise Massenanfall an Erkrankten)
	Üben größerer Einsatzlagen mit Einbeziehung von Hilfsorganisationen
	Umgang mit der Presse und Kommunikationsstrategien
Gefährdungsbeurteilung und Prävention	Gesetzliche Grundlagen, Regelwerke, Vorschriften (Internationale Gesundheitsvorschriften)
	Neu auftretende Infektionskrankheiten (Emerging Infectious Diseases) und globale Infektionskrankheiten
	Erhalt von strukturierten und validen Informationen über aktuelle und dynamische Lagen
	Übertragungswege an Bord auf hoher See
	Übersichtslisten zu möglichen Infektionen und deren Übertragungswegen, die für Laien verständlich sind
	Relevante Vektoren für den maritimen Bereich und die Kontrolle von Vektoren
	Wissen über chemische, biologische, radiologische und nukleare Gefahren (CBRN)
	Schemata zur Einschätzung von Gefahren und Entscheidungsfindung
	Sensibilisierung und Abbau von Ängsten unter Mitarbeitenden
	Auswahl von und Umgang mit persönlicher Schutzausrüstung
	Praktische Übungen im Vollschutz

### Dokumentenanalyse

Begleitend zu den Interviews wurde eine Dokumentenanalyse von vorhandenen Notfallplänen und Standardarbeitsanweisungen (SOPs) durchgeführt. Hierzu wurden IGV-Notfallpläne mitsamt den dazugehörigen Dokumenten gesichtet. Dazu gehörten unter anderem:

- Kommunikation (Meldeketten, Kontaktdaten, Durchsagetexte etc.),
- Übersichtspläne der Häfen (Terminals etc.),
- Hygiene‑/Desinfektionspläne,
- Materialvorhaltungen/persönliche Schutzausrüstung,
- Protokollvorlagen.

Gemeinsam mit den IGV-Notfallplänen wurden insgesamt 177 einzelne Dokumente anhand unterschiedlicher Kriterien (Aktualität, Verlauf, Herausgabe und Freigabe) beurteilt. Anschließend wurden die Dokumente nach den IGV-Kernkapazitäten klassifiziert. Die Analyse ergab, dass der Umfang und der Detailgrad der vorhandenen Notfallpläne je nach Standort variierten. Einige Pläne befanden sich noch in der Entwicklung. Zudem wurden einige Lücken bezüglich der IGV-Notfallplanung festgestellt. Die untersuchten Dokumente dienten im weiteren Projektverlauf als Grundlage für die Erstellung von generischen Versionen.

## Entwicklung eines generischen Prozesses zur Bewältigung von infektiologischen Ereignissen

### Runde Tische und Workshops

**Abb. 2:**
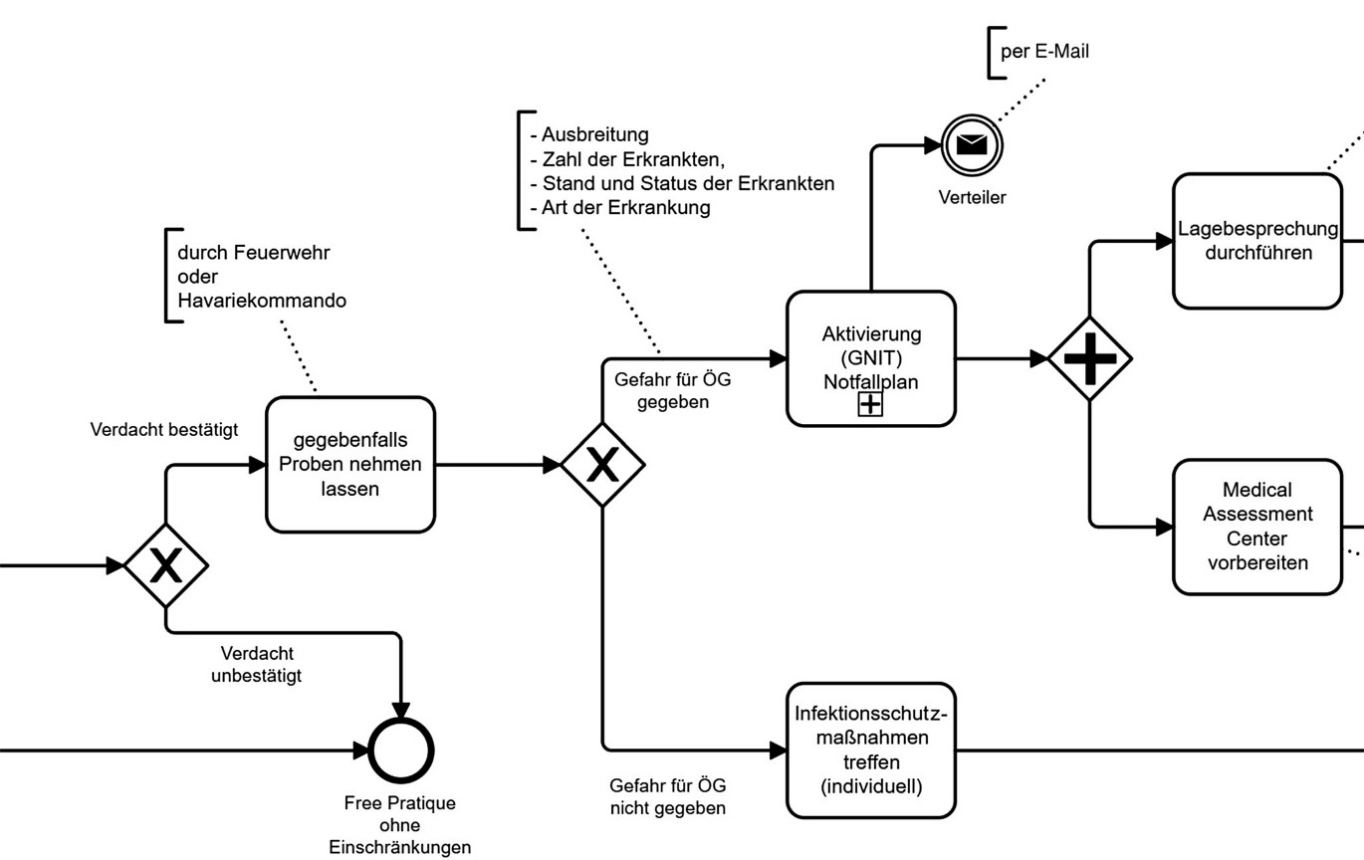
Ausschnitt aus einem exemplarischen Handlungsalgorithmus, basierend auf Prozessschilderungen im Interview mit einem Hafenärztlichen Dienst. *ÖG* Öffentliche Gesundheit; *GNIT* Gesundheitliche Notlage internationaler Tragweite

Ein zentrales Ziel des Projektes bestand darin, bestehende Abläufe zu analysieren und zu harmonisieren. Im Rahmen der oben beschriebenen Interviews wurden die Zuständigkeiten und Tätigkeiten der Akteure erhoben und mithilfe von Diagrammen zur Prozessmodellierung visuell dargestellt (Abb. 2). Diese Prozessdarstellungen bildeten die Grundlage für einen strukturierten Austausch an runden Tischen in den jeweiligen Häfen, bei denen sie gemeinsam mit den lokalen Beteiligten validiert und diskutiert wurden.

Basierend auf den Erkenntnissen aus den 5 Standorten wurde ein erster generischer Prozess für den Hafenärztlichen Dienst konzipiert, der im Verlauf des Projektes weiterentwickelt wurde. Ein Meilenstein dazu war ein gemeinsamer Workshop mit den Hafenärztlichen Diensten und dem RKI im Juli 2024 in Hamburg. Dabei wurde u. a. diskutiert, an welchen Stellen eine Ergänzung durch Standardarbeitsanweisungen sinnvoll ist. Im September 2024 wurde die weiterentwickelte Version im Rahmen der Lübecker Hafentage in einem größeren und interdisziplinären Kreis diskutiert. Beteiligte waren vor allem Fachkräfte aus Gesundheitsämtern, die mit den Aufgaben des HÄD betraut sind und unter anderem auch in kleineren Häfen tätig sind.

### Beschreibung des finalen generischen Prozesses

**Abb. 3:**
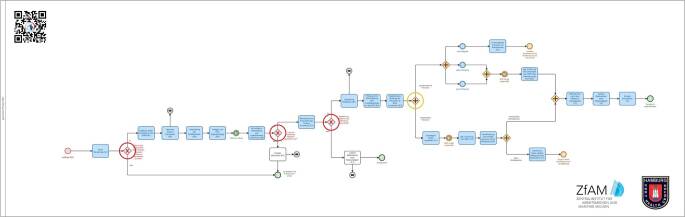
Beispielhafte Darstellung eines generischen Prozesses zur Bewältigung von infektiologischen Ereignissen am Hafen. Veranschaulichung der Komplexität. *Rote Markierungen* zeigen exklusive Gateways (entweder/oder), die *gelbe Markierung* zeigt ein paralleles Gateway (und/oder). Eine hochauflösende und detaillierte Version findet sich auf der Projekt-Homepage www.projekt-gesa.de

Eine detaillierte Version des entwickelten generischen Prozesses befindet sich auf der Projekt-Homepage (www.projekt-gesa.de). Die Komplexität der Darstellung eines solchen Prozesses wird in Abb. 3 exemplarisch veranschaulicht. Den Startpunkt für den generischen Prozess stellt in der Regel eine auffällige Seegesundheitserklärung (Maritime Declaration of Health – MDH) dar, die über das zentrale elektronische Meldesystem für den Seeverkehr, das National Single Window (NSW), gemeldet wird. Grundsätzlich verzweigt sich der Prozess an folgenden 3 exklusiven Gateways (entweder/oder) mit richtungsweisenden Leitfragen:

1. Verdacht: Gibt es den begründeten Verdacht eines relevanten Infektionsgeschehens an Bord?
2. Verifizierung: Liegt ein relevantes Infektionsgeschehen vor?
3. Eskalation: Besteht eine Gefahr für die öffentliche Gesundheit?

In der höchsten Eskalationsstufe werden ab einem vierten parallelen Gateway (und/oder) weitere notfallmedizinische und gesundheitsbehördliche Prozesse bei bereits symptomatischen sowie bei symptomlosen, aber ansteckungsverdächtigen Personen eingeleitet. Notfallmedizinisch liegt ein Augenmerk auf der Sichtung/Begutachtung und Priorisierung erkrankter Personen zur weiteren Behandlung und dem Transport in medizinische Einrichtungen. Der ÖGD führt im Rahmen des Kontaktpersonenmanagements Befragungen krankheitsverdächtiger Personen durch. Diese und weitere Infektionsschutzmaßnahmen werden auf Grundlage des Infektionsschutzgesetzes (IfSG), der IGV sowie weiterer Gesetze und Richtlinien angeordnet und überwacht.

Ein weiterer Fokus bei der Prozessentwicklung lag auf der Identifikation und Beschreibung von Schnittstellen zwischen den Akteuren. So sind eine rechtzeitige und detaillierte Informationsübermittlung sowie stetige Kommunikation von essenzieller Bedeutung. Dies ist insbesondere für Akteure relevant, die im Rahmen ihrer regulären Tätigkeit an Bord gehen und somit frühzeitig über potenzielle Risiken informiert werden müssen, um sich entsprechend schützen zu können.

### Validierung des Prozesses anhand eines Planspiels

**Abb. 4:**
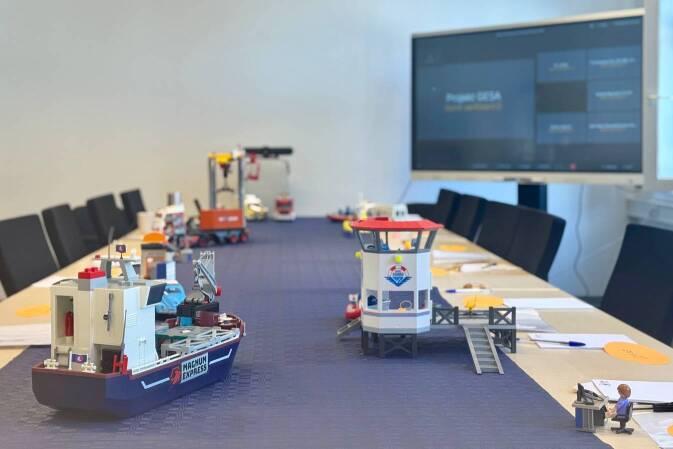
Durchführung eines Planspiels mithilfe von Spielzeugfiguren. Die Figuren verdeutlichen die jeweiligen einzunehmenden Rollen

Zur Erprobung, Beurteilung und Optimierung des entwickelten generischen Prozesses wurde ein Planspiel konzipiert. Dieses wurde im Dezember 2024 mit Akteuren des Hamburger Hafens durchgeführt. Vertreterinnen und Vertreter der anderen Hafenärztlichen Dienste und des RKI nahmen als Beobachtende an dem Planspiel teil. Als Szenario wurde das Vorkommen eines Meningitis-Infektionsfalls an Bord eines Containerschiffes simuliert. Der Einsatz von Spielzeugfiguren ermöglichte die Entwicklung einer visuell ansprechenden und spielerischen Herangehensweise. Diese erleichterte das Verständnis des zugrunde liegenden Szenarios und der Rollen der verschiedenen Akteure (Abb. 4).

Während der Übung wurden verschiedene Aspekte des Notfallmanagements durchgespielt, darunter die schnelle Beurteilung der Lage, die medizinische Versorgung der erkrankten Personen sowie die Identifikation und Nachverfolgung möglicher Kontaktpersonen. Mit dem Ziel einer zeitnahen und präzisen Kommunikation zwischen den verschiedenen Akteuren wurde ein Fokus auf die Kenntnis bestehender Meldewege, Verantwortlichkeiten und die Nutzung zeitgemäßer Kommunikationsmittel gelegt. Gleichzeitig wurden durch die Teilnehmenden des Planspiels konkrete Verbesserungsvorschläge geäußert, beispielsweise das Vorhalten von persönlicher Schutzausrüstung und Desinfektionsmitteln direkt am Terminal sowie die Möglichkeit einer orientierenden telemedizinischen Diagnostik durch den Hafenärztlichen Dienst, beispielsweise anhand von Fotos mit Hautbefunden.

## Wissenstransfer

### Durchführung einer Pilotschulung zum Notfallmanagement im Hafenärztlichen Dienst

Auf Basis der in den Interviews geäußerten Bedarfe wurde ein konkretes Schulungsformat entwickelt. Hierzu wurden besonders oft genannte Themen wie Grundwissen zu den IGV und spezifische Maßnahmen und Abläufe im Hafenärztlichen Dienst aufgegriffen. Im April 2025 wurde in Kooperation mit der Akademie für Öffentliches Gesundheitswesen (AÖGW) ein hybrides Seminar in Hamburg zum Thema „Notfallmanagement im Hafenärztlichen Dienst bei übertragbaren Krankheiten“ durchgeführt. Zielgruppe waren Mitarbeitende der Hafenärztlichen Dienste sowie Beschäftigte in Gesundheitsämtern mit Bezug zu Hafenstandorten. Insgesamt nahmen 49 Personen teil, davon 34 online.

Zentrale Bestandteile der Schulung waren eine praxisnahe Einführung in die IGV durch einen Referenten des RKI, die Vorstellung von guten Praxisbeispielen am IGV-Hafen Hamburg und ein Planspiel zur interaktiven Vermittlung des generischen Prozesses. Der Fokus lag dabei insbesondere auf den Aufgaben des ÖGD und der Schnittstellenkoordination zwischen den verschiedenen Akteuren im Hafen. Die Online-Teilnehmenden wurden aktiv über das Umfrage- und Abstimmungstool „Mentimeter“ (https://www.mentimeter.com/) in das Planspiel eingebunden.

Die Veranstaltung wurde insgesamt sehr positiv bewertet. Besonders großen Zuspruch erhielt auch das interaktive Planspiel. Insbesondere hat die Schulung die angestrebte Zielgruppe erreichen können: Die Hälfte der Teilnehmenden kam aus dem ärztlichen Bereich, weitere 37 % waren als Hygienekontrolleurinnen und Hygienekontrolleure tätig. 2 Drittel der Teilnehmenden waren bereits beruflich in einem Hafen eingebunden, während 14 % künftig Aufgaben in diesem Bereich übernehmen werden.

### Entwicklung eines Schulungsvideos zum generischen Prozess

In Zusammenarbeit mit einem Mediengestalter wurde ein Schulungsvideo produziert, das die zentralen Maßnahmen innerhalb des generischen Prozesses veranschaulicht. Hierzu wurde zunächst ein Skript auf Basis des Prozesses entwickelt und mithilfe von KI-generierten Szenen umgesetzt. Durch die visuelle Darstellung sollen die Abläufe besser verständlich werden und ihre praktische Umsetzung erleichtert werden. Das Video soll gleichzeitig weiteren Akteuren am Hafen eine thematische Orientierung geben und sie für die Aufgabenbereiche des Hafenärztlichen Dienstes sensibilisieren.

### Erstellung eines Handbuchs für den Hafenärztlichen Dienst

Zur Verstetigung der Projektergebnisse wurde auf Basis des generischen Prozesses ein Handbuch mitsamt eines individuell anpassbaren Notfallplans erstellt. Dieser wurde als Vorlage für das Notfallmanagement bei übertragbaren Krankheiten im Hafenärztlichen Dienst konzipiert. Der Prozess wurde hierbei in schriftlicher Form dargelegt und um zugehörige SOPs und Dokumente ergänzt. Zu den Dokumenten zählen unter anderem Checklisten, welche die Mitnahme von Material und persönlicher Schutzausrüstung regeln, sowie Protokolle über Ereignisse, Anamnese- und Untersuchungsbögen und Kommunikationsschemata. Das Handbuch kann als Blaupause für einen Notfallplan dienen, da die SOPs und Vorlagen flexibel an die spezifischen Gegebenheiten der Häfen angepasst werden können. Ein Peer-Review-Verfahren durch alle Projektbeteiligten vor der finalen Veröffentlichung des Handbuchs sollte dabei sicherstellen, dass die Perspektiven der jeweiligen Häfen berücksichtigt werden und Verständlichkeit und Anwendbarkeit gewährleistet sind.

## Schlussfolgerungen

Das Projekt GESA schafft die Grundlage für eine verbesserte Koordination und Standardisierung der Maßnahmen beim Infektionsmanagement in deutschen Häfen und stellt durch die Entwicklung des Schulungskonzeptes einen wichtigen Schritt zur Umsetzung der Internationalen Gesundheitsvorschriften sowie zur Stärkung der maritimen Gesundheitssicherheit in Deutschland dar. Die initiale Analyse des Ist-Zustands in den 5 IGV-Häfen ermöglichte Einblicke in die bestehenden Strukturen und Prozesse. Im Rahmen der durchgeführten Interviews mit den relevanten Akteuren sowie der Dokumentenanalyse bestehender Notfallpläne konnten Schwachstellen und Verbesserungspotenziale identifiziert werden. Die enge Zusammenarbeit und der Austausch zwischen den beteiligten Akteuren haben gezeigt, dass eine standardisierte Vorgehensweise möglich ist, sofern diese spezifisch an die jeweiligen Hafenstrukturen angepasst werden kann. Durch die Veröffentlichung der Ergebnisse kann auch der ÖGD an anderen Häfen in Deutschland von den während des GESA-Projektes erarbeiteten Erkenntnissen profitieren.

### Kollaborative Konzeptentwicklung

Im Hinblick auf die Prävention und Vorbereitung auf Infektionsausbrüche an Bord sowie in Häfen sind die Entwicklung und regelmäßige Aktualisierung von Notfallplänen von zentraler Relevanz. Zudem sind eine enge Zusammenarbeit und Kommunikation aller beteiligten Akteure erforderlich [10]. Die Durchführung von runden Tischen und Workshops mit den HÄD, dem RKI und weiteren relevanten Akteuren stellte daher einen wichtigen Bestandteil in der Erarbeitung des Konzeptes dar. Diese Formate ermöglichten nicht nur die Rückkopplung und Weiterentwicklung der zuvor identifizierten Prozesse, sondern förderten auch den interdisziplinären Austausch. In allen beteiligten Hafenstandorten wurde angeregt, die runden Tische künftig eigenständig fortzuführen. So wurde die Möglichkeit gegeben, einen kontinuierlichen, praxisnahen Dialog zwischen allen beteiligten Akteuren zu etablieren und damit die Zusammenarbeit langfristig zu stärken. Zudem war es anhand des Feedbacks aus dem Planspiel möglich, die Stärken und Schwächen des generischen Prozesses zu identifizieren und diesen zu optimieren.

Bei der Erarbeitung der standardisierten Prozesse war es von entscheidender Bedeutung, die jeweiligen Gegebenheiten der Häfen zu berücksichtigen. Es konnte festgestellt werden, dass jeder Hafen standortspezifische Besonderheiten aufweist. Diese Besonderheiten beziehen sich beispielsweise auf die lokale Gestaltung der Hafenanlagen, den Frachtumschlag, das Passagieraufkommen und die personellen Ressourcen der Akteure. Bei der Entwicklung generischer Prozesse und SOPs war es entscheidend, die spezifischen Gegebenheiten einzelner Häfen zu berücksichtigen und jeweils anpassbare Musterdokumente zu erstellen. Zudem wurde der Aspekt der Übertragbarkeit auf weitere Häfen, die nicht nach den IGV benannt sind, berücksichtigt.

### Technologische Unterstützung und Digitalisierung

Die Durchführung einer hybriden Pilotschulung ermöglichte es, die Reichweite zu erhöhen und eine niedrigschwellige Teilnahmemöglichkeit zu bieten. Durch die gezielte Ausarbeitung des Planspiels für den hybriden Einsatz unter Einbindung des Online-Umfragetools konnten auch die Online-Teilnehmenden in den praktischen Teil des Seminars eingebunden werden. Die Entwicklung von hybriden oder digitalen Übungsformaten kann als Ansatz betrachtet werden, um den ermittelten Bedarf in der Aus- und Fortbildung zu adressieren, insbesondere im Hinblick auf die Übung von Abläufen und Schnittstellen zwischen den Akteuren. Im Bereich der Stabsarbeit bietet der Einsatz digitaler Übungsplattformen als Alternative zu Übungen in Präsenz bereits Potenzial [11]. Obgleich Übungen vor Ort zur praxisnahen Beübung von Einsatzkonzepten sowie in Zusammenarbeit verschiedener Akteure unabdingbar sind, bieten digitale Übungsformate die Möglichkeit, die mit derartigen Übungen in Präsenz einhergehenden Kosten und Ressourcen zu senken und eine höhere Übungsfrequenz zu gewährleisten.

Darüber hinaus kann die Nutzung digitaler Werkzeuge zur Surveillance und Kommunikation die Reaktionsfähigkeit des Öffentlichen Gesundheitsdienstes in einer infektiologischen Gefahrenlage signifikant erhöhen. Der Einsatz von künstlicher Intelligenz (KI) und algorithmisch gesteuerten Systemen zur Lageeinschätzung und Bewertung epidemiologischer Risiken bietet in diesem Zusammenhang ein hohes Potenzial [12], das in diesem Projekt noch nicht ausgeschöpft werden konnte. Die Entwicklung von Algorithmen auf Basis globaler Datensätze könnte eine frühzeitige Erkennung epidemiologischer Zusammenhänge ermöglichen und somit die Erstellung fundierter Risikoeinschätzungen durch den Hafenärztlichen Dienst unterstützen. Ein erster Ansatz hierzu befindet sich momentan am Hamburg Port Health Center in Entwicklung. Die Integration neuer Technologien in bestehende Abläufe stellt somit einen vielversprechenden Ansatz dar, der einer weiteren Erforschung sowie einer gezielten Weiterentwicklung bedarf.

### Nachhaltigkeit und Ausblick

Eine zentrale Herausforderung bestand darin, die Nachhaltigkeit des Projektes nach Abschluss sicherzustellen und dessen Ergebnisse langfristig zu sichern. Durch das entwickelte Schulungscurriculum konnte hierbei die Möglichkeit eines stetigen Wissenstransfers etabliert werden. So sollen die Projektergebnisse in Fortbildungsprogramme eingebunden werden, beispielsweise über die AÖGW. Perspektivisch wollen die Verbundpartner in Zusammenarbeit mit der AÖGW ein E‑Learning-Modul entwickeln, das darauf abzielt, die vermittelten Inhalte nachhaltig zu verankern und die kontinuierliche Weiterbildung von Mitarbeitenden in zuständigen Gesundheitsbehörden zu fördern. Der Fokus des E‑Learning-Moduls wird auf der Vermittlung der theoretischen Inhalte (rechtliche Rahmenbedingungen, gute Praxisbeispiele Hamburg, generischer Prozess) im Sinne eines Blended-Learning-Ansatzes als Vorbereitung auf zukünftige Präsenzschulungen liegen, sodass sich die Schulungen vor Ort verstärkt der praktischen Umsetzung in Form des Planspiels und der persönlichen Interaktion der Teilnehmenden widmen können.

Das Handbuch dient dazu, die erarbeiteten standardisierten Prozesse sowie SOPs nachhaltig umzusetzen. Die Ergebnisse und Arbeitsmaterialien sollen über die Projekt-Homepage (https://projekt-gesa.de/) öffentlich zugänglich gemacht werden. Plattformen wie „Agora“ als Kollaborationsplattform für den ÖGD oder der Arbeitskreis der Küstenländer für Schiffshygiene (AkKü) mit seiner Web-Präsenz bieten zusätzlich die Möglichkeit, eine kontinuierliche Bereitstellung der Materialien zu gewährleisten. Regelmäßige Überprüfungen und Anpassungen der Prozesse und SOPs sind notwendig, um sicherzustellen, dass die Maßnahmen den aktuellen Anforderungen und Best Practices entsprechen. Um sicherzustellen, dass alle Beteiligten mit dem Konzept vertraut sind und im Ernstfall effektiv handeln können, sollten regelmäßige Schulungen und Übungen durchgeführt werden. Perspektivisch sollte das im Projekt entwickelte standardisierte Konzept in Form von Vollübungen sowie unter realen Bedingungen evaluiert werden.

## Fazit

Die erstellten Arbeitsmaterialien des Projektes ermöglichen es jedem Hafen in Deutschland, einen maßgeschneiderten Notfallplan zum Management von Infektionsgeschehen zu erstellen oder zu überarbeiten, der auf einer abgestimmten, standardisierten Grundlage basiert. Es ist wichtig, dass das Konzept nicht nur kurzfristig implementiert, sondern auch langfristig aufrechterhalten wird. Dies erfordert neben den Maßnahmen des Wissenstransfers eine Verstetigung durch eine finanzielle Absicherung sowie klare Verantwortlichkeiten innerhalb der zuständigen Behörden. Besonders entscheidend ist dabei eine feste Verankerung im öffentlichen Gesundheitswesen, da nur sie den notwendigen Rahmen für langfristige strategische Maßnahmen schaffen kann. Nur so können das Fortbestehen und die Weiterentwicklung abgestimmter Entscheidungsprozesse gewährleistet und die notwendigen Ressourcen bereitgestellt werden, um so Zuständigkeiten klar zu definieren und Kooperationen zu stärken.
